# Identification of *POMC* Exonic Variants Associated with Substance Dependence and Body Mass Index

**DOI:** 10.1371/journal.pone.0045300

**Published:** 2012-09-17

**Authors:** Fan Wang, Joel Gelernter, Henry R. Kranzler, Huiping Zhang

**Affiliations:** 1 Department of Psychiatry, Yale University School of Medicine, New Haven, Connecticut, United States of America; 2 Department of Genetics, Yale University School of Medicine, New Haven, Connecticut, United States of America; 3 Department of Neurobiology, Yale University School of Medicine, New Haven, Connecticut, United States of America; 4 Veterans Affairs (VA) Medical Center, VA Connecticut Healthcare System, West Haven, Connecticut, United States of America; 5 Department of Psychiatry, University of Pennsylvania Perelman School of Medicine and VISN4 Mental Illness Research, Education and Clinical Center (MIRECC), Philadelphia Veterans Affairs Medical Center (VAMC), Philadelphia, Pennsylvania, United States of America; McLean Hospital/Harvard Medical School, United States of America

## Abstract

**Background:**

Risk of substance dependence (SD) and obesity has been linked to the function of melanocortin peptides encoded by the proopiomelanocortin gene (*POMC*).

**Methods and Results:**

*POMC* exons were Sanger sequenced in 280 African Americans (AAs) and 308 European Americans (EAs). Among them, 311 (167 AAs and 114 EAs) were affected with substance (alcohol, cocaine, opioid and/or marijuana) dependence and 277 (113 AAs and164 EAs) were screened controls. We identified 23 variants, including two common polymorphisms (rs10654394 and rs1042571) and 21 rare variants; 12 of which were novel. We used logistic regression to analyze the association between the two common variants and SD or body mass index (BMI), with sex, age, and ancestry proportion as covariates. The common variant rs1042571 in the 3′UTR was significantly associated with BMI in EAs (Overweight: *P*_adj_ = 0.005; Obese: *P*_adj_ = 0.018; Overweight+Obese: *P*_adj_ = 0.002) but not in AAs. The common variant, rs10654394, was not associated with BMI and neither common variant was associated with SD in either population. To evaluate the association between the rare variants and SD or BMI, we collapsed rare variants and tested their prevalence using Fisher’s exact test. In AAs, rare variants were nominally associated with SD overall and with specific SD traits (SD: *P*_FET,1df_ = 0.026; alcohol dependence: *P*_FET,1df_ = 0.027; cocaine dependence: *P*_FET,1df_ = 0.007; marijuana dependence: *P*_FET,1df_ = 0.050) (the *P*-value from cocaine dependence analysis survived Bonferroni correction). There was no such effect in EAs. Although the frequency of the rare variants did not differ significantly between the normal-weight group and the overweight or obese group in either population, certain rare exonic variants occurred only in overweight or obese subjects without SD.

**Conclusion:**

These findings suggest that *POMC* exonic variants may influence risk for both SD and elevated BMI, in a population-specific manner. However, common and rare variants in this gene may exert different effects on these two phenotypes.

## Introduction

Substance dependence (SD) and obesity are two prevalent health problems. They are highly heritable, but the underlying genetic mechanisms are, for the most part, not well understood. Since both substances of abuse and food have rewarding properties, it is possible that overlapping reward pathways in the brain are involved in SD and obesity development. Deficits in neural reward responses and alterations in reward homeostasis are thought to be a common mechanism for obesity and SD [1]. A number of studies have demonstrated an inverse relationship between BMI and SD [2], [3], [4], [5], [6]. Although other studies failed to support this finding [7], [8], [9], it is still of interest to identify genetic variation contributing to SD, obesity or both. Identification of variation in genes participating in the common reward pathway for SD and obesity could help in the design of hypothesis-guided therapies to treat both conditions.

**Figure 1:**
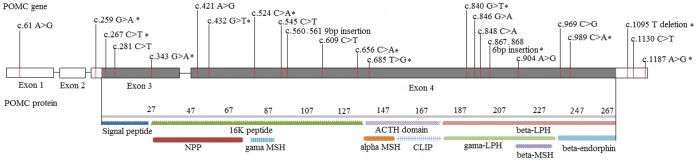
Schematic genomic structure of *POMC* and locations of identified variants. Asterisk “*” indicates that the variant was newly identified.

Both obesity and SD can result from dysfunction of melanocortin peptides, which are components of the hypothalamic-pituitary-adrenal (HPA) axis. Melanocortin peptides are encoded by the proopiomelanocortin (POMC) gene (*POMC*) on chromosome 2p23 [10], [11]. They are derived from POMC after extensive tissue-specific cleavage and include as many as 10 functionally different peptides such as adrenocorticotropic hormone (ACTH), α-, β-, and γ-melanocyte-stimulating hormone (MSH), β- and γ-lipotropin, corticotropin-like intermediate peptide (CLIP) and β-endorphin (**Figure 1**). Melanocortin peptides play important roles in pain [12], energy homeostasis [13], melanocyte stimulation [14] and immune modulation [15]. Of these peptides, ACTH and β-endorphin have been implicated in craving for substances of abuse. Plasma ACTH and β-endorphin levels were significantly lower in heavy drinkers than in non-drinkers [16]. Further, a significant decrease in plasma levels of ACTH and β-endorphin was shown in abstinent alcoholics [17]. The decreased level of β-endorphin in alcoholics and the increased release of β-endorphin after alcohol consumption support the theory of β-endorphin deficiency in alcoholism [18]. Melanocortin peptides and their receptors are also involved in hormonal regulation of pigmentation, weight maintenance, adrenal function and exocrine gland secretion. Animal studies elucidated a dual role of α-MSH in regulating food intake and influencing hair pigmentation [18]. Augmented ACTH and β-lipotrophin secretion was shown in patients with obesity [19]. Thus, functional alterations in melanocortin peptides due to variation in *POMC* may predispose to SD and obesity, as well as other traits.

Four published studies have provided evidence that *POMC* variants can regulate SD risk. Xuei *et al* [20] reported that two single nucleotide polymorphisms (SNPs) in *POMC* intron 1 were associated with opioid dependence (OD) in a family-based study. Racz *et al* [21] found that a two-SNP haplotype in *POMC* was associated with alcohol dependence (AD) in females. We conducted both family- and population-based studies (3,088 subjects) and found that variants in *POMC* promoter and intronic regions conferred vulnerability to multiple forms of SD [22]. Recently, a genome-wide association study revealed that variants in *POMC* were nominally associated with AD in African Americans (AAs) [23].

*POMC* variation also influences traits such as obesity and hair pigmentation. Loss-of-function mutations [e.g., a homozygous mutation (C3804A) in exon 2 (now exon 3) and a compound heterozygote for two mutations (G7013T and 1 bp-deletion C7133Δ) in exon 3 (now exon 4)], resulting in ACTH and α-MSH deficiency, caused severe early-onset obesity, adrenal insufficiency and red hair pigmentation [24]. Linkage analysis confirmed the trend (maximum LOD at D2S2337 = 2.03) towards linkage between polymorphic markers around *POMC* and obesity [25]. Heterozygosity for any of the three non-synonymous mutations (G3834C or Ser7Thr and C3840T or Ser9Leu within *POMC* signal peptide, and C7406G or Arg236Gly within β-endorphin) and a 9-bp insertion/deletion polymorphism (−/AGCAGCGGC or rs10654394) were found in obese children [26]. The 9-bp-insertion allele was also associated with elevated serum leptin levels [27]. However, Echwald *et al* [28] did not find an association between *POMC* exonic variants (including the 9-bp insertion/deletion polymorphism) and early-onset obesity is a sample of 156 obese Caucasians and 380 healthy controls.

To date, *POMC* exons have not been sequenced in a large sample. Some unknown exonic variants may have not been identified and their association with diseases such as SD is waiting to be studied. In the present study, we sequenced all *POMC* exons in a relatively large case-control sample. The identified common and rare exonic variants were analyzed for the association with SD and/or BMI.

## Methods

### Study Subjects

Subjects were recruited from three sites in the United States: the University of Connecticut Health Center (Farmington, Connecticut), Yale University School of Medicine (APT Foundation, New Haven, Connecticut), and the Medical University of South Carolina (Charleston, South Carolina). The study protocol was approved by each local institutional review board (the Institutional Review Board of the University of Connecticut, the Yale University Human Research Protection Program, and the Institutional Review Board for Human.

Research of the Medical University of South Carolina), and written informed consent was obtained from each subject. Subjects were interviewed by trained interviewers using the Semi-Structured Assessment for Drug Dependence and Alcoholism (SSADDA) instrument [29], [30], which yielded diagnoses for lifetime substance dependence (SD) and other psychiatric traits based on the criteria of the Diagnostic and Statistical Manual of Mental Disorders, 4^th^ edition (DSM-IV) [31].

A total of 588 subjects [280 African Americans (AAs) and 308 European Americans (EAs)] were selected for this study. Among them, 311 (167 AAs and 114 EAs) were affected with substance (alcohol, cocaine, opioid and/or marijuana) dependence (AD, CD, OD and/or MjD, respectively) and 277 (113 AAs and 164 EAs) were screened controls. Of the 167 AA cases, 131 (78.4%) had AD, 118 (70.7%) had CD, 24 (14.4%) had OD, and 68 (40.7%) had MjD. Of the 144 EA cases, 112 (98.2%) had AD, 85 (74.5%) had CD, 50 (43.9%) had OD, and 44 (38.6%) had MjD. Control subjects were not affected with AD, CD, OD and/or MjD. Major psychotic disorders such as schizophrenia and bipolar disorders were exclusion criteria for both groups. Among the 588 subjects, 51 AA cases, 40 AA controls, 15 EA cases and 41 EA controls were included in our previous study [22]. Additionally, self-reports for current height and weight were obtained, and were used to estimate BMI [i.e., weight in kg/(height in meters)^2^]. According to the World Health Organization (WHO) criteria, three subjects (BMI ± S.D.:  = 18.4±0.1) were classified as underweight (BMI <18.5), 195 subjects (BMI ± S.D.: 22.4±1.6) were classified as normal weight (BMI: 18.5–24.9), 212 subjects (BMI ± S.D.: 27.1±1.4) were classified as overweight (BMI: 25.0–29.9), and 178 subjects (BMI ± S.D.: 35.5±5.1) were classified as obese (BMI ≥30). The mean ± S.D. of BMI for AA cases was 29.0±6.5, which was not significantly different from that of AA controls (29.2±7.1) (T  = 0.248, *P*  = 0.805). The mean ± S.D. of BMI for EA cases was 28.3±5.3, which was significantly higher than that of EA controls (26.1. ±5.4) (T  = 3.613, *P*  = 0.0004). Detailed demographic and clinical information on the study samples is presented in **Table 1** **.**

**Table 1:** Characteristics of case and control subjects.

	African Americans (AAs)	European Americans (EAs)	AAs + EAs
	(n = 280)	(n = 308)	(n = 588)
SD cases (n)	167	144	311
AD, n (%)	131 (78.4%)	112 (98.2%)	243 (78.1%)
CD, n (%)	118 (70.7%)	85 (74.5%)	203 (65.3%)
OD, n (%)	24 (14.4%)	50 (43.9%)	74 (23.8%)
MjD, n (%)	68 (40.7%)	44 (38.6%)	82 (26.4%)
Sex, Male (%)	92 (55.1%)	96 (66.7%)	188 (60.1%)
Age, years (mean ± S.D.)	41±9	40±11	40±10
BMI (mean ± S.D.)	29.0±6.5	28.3±5.3	28.7±5.9
Underweight (BMI <18.5), n (%)	0 (0%)	0 (0%)	0 (0%)
Normal weight (BMI: 18.5–24.9), n (%)	45 (29.6%)	39 (27.1%)	84 (27.0%)
Overweight (BMI: 25–29.9), n (%)	62 (37.1%)	61 (42.4%)	123 (39.5%)
Obese (BMI ≥30), n (%)	60 (33.3%)	44 (30.5%)	104 (33.5%)
Controls (n)	113	164	277
Sex, Male (%)	75 (66.4%)	100 (61.0%)	175 (63.2%)
Age, years (mean ± S.D.)	38±14	38±15	38±15
BMI (mean ± S.D.)	29.2±7.1	26.1±5.4	27.4±6.3
Underweight (BMI <18.5), n (%)	0 (0%)	3 (1.8%)	3 (1.1%)
Normal weight (BMI: 18.5–24.9), n (%)	31 (27.4%)	80 (48.8%)	111 (40.1%)
Overweight (BMI: 25–29.9), n (%)	42 (37.2%)	47 (28.7%)	89 (32.1%)
Obese (BMI ≥30), n (%)	40 (35.4%)	34 (20.7%)	74 (26.7%)

SD: substance (alcohol, cocaine, opioid and/or marijuana) dependence.

AD: alcohol dependence; CD: cocaine dependence; OD: opiate dependence; MjD: marijuana dependence.

BMI: body mass index (kg/m^2^).

### Sequencing

*POMC* spans about an 8 kb genomic region (Chromosome 2: 25,383,722–25,391,772) and contains four exons. It encodes a protein of 247 amino acids. We designed primers for polymerase chain reactions (PCRs) according to *POMC* DNA sequence obtained from the Ensembl database (ENST00000380794). PCR products were treated with ExoSAP-IT PCR clean-up reagents (USB Corporation, Cleveland, OH, USA). DNA sequencing was performed on Applied Biosystems 3730 capillary instruments (Applied Biosystems, Foster City, CA, USA) using reagents in the BigDye Direct Cycle Sequencing Kit (Applied Biosystems, Foster City, CA, USA) at the Yale Keck core facility. DNA sequencing was first conducted in the forward direction using forward primers, and then DNA sequences potentially harboring variants were sequenced in the reverse direction using reverse primers for validation. PCR and sequencing primer sequences for each exon are presented in **Supporting Information Table S1** and PCR conditions for amplifying the four exons are listed in **Supporting Information Table S2.** DNA sequencing raw data were first analyzed using the Sequence Scanner v1.1 (Applied Biosystems, Foster City, CA, USA). Sequencing chromatograms were then loaded into the program CodonCode Aligner v3.7.1 (CodonCode Corporation, Dedham, MA, USA) to determine homozygous and heterozygous calls.

### Statistical Analysis

To verify the self-reported race, we used a Bayesian model-based clustering method implemented in the program STRUCTURE [32], [33]. We estimated the African and European ancestry proportions of individual subjects, based on the genotype data of 41 ancestry informative markers (AIMs), including 36 short tandem repeat markers and five SNPs, as described previously [34], [35]. Subjects with ancestry proportion scores ≥0.50 were grouped as African Americans (AAs), and those with ancestry proportion scores <0.50 were grouped as European Americans (EAs). These two distinct groups were highly concordant with self-reported AA and EA group membership. In this study, we analyzed the association of the identified *POMC* exonic variants with: (1) SD overall; (2) specific forms of SD (i.e., AD, CD, OD, or MjD); and (3) BMI. BMI was converted into four categorical traits [underweight (BMI <18.5), normal weight (BMI: 18.5–24.9), overweight (BMI: 25.0–29.9), and obese (BMI ≥30)] as described in **Table 1** **.** Three models were considered (Model 1: overweight group *vs*. normal weight group; Model 2: obese group *vs*. normal weight group; Model 3: overweight + obese group *vs*. normal weight group).

We first analyzed the association of the two identified *POMC* common variants with SD or BMI. Tests for Hardy-Weinberg equilibrium (HWE) were conducted in AA and EA control subjects. Allelic association analysis was performed using Pearson’s 2×2 contingency table Chi-square (χ^2^) tests. Odds ratios (ORs) and 95% confidence intervals (CIs) were estimated using χ^2^ tests. To adjust for multiple testing, permutation tests were performed 10,000 times to calculate empirical *P* values. Multivariate logistic regression analysis was used to calculate *P* values, with covariance adjustment for sex, age, and ancestry proportion (for binary SD traits, BMI was taken as an additional covariate; for BMI in three models, SD status was taken as an additional covariate). The above association analyses were implemented by the program PLINK v1.07 [36]. Haplotypes of the two identified common variants were constructed using program Haploview v4.2. [37], and haplotype-based association analysis was performed using PLINK. Statistical power analysis was conducted by program PS (Power and Sample size Calculations, version 3.0.43) [38].

We also analyzed the association of identified *POMC* rare variants with SD or BMI (in three models). Rare variants were defined as SNP markers with a minor allele frequency (MAF) less than 1%, consistent with previous studies [39], [40]. We found 20 variants with MAFs less than 1% and one variant with a MAF of 0.011. All of these 21 variants were included in the aggregate analysis using Fisher exact tests. To increase statistical power, we used a collapsing method in which alleles of all identified rare variants were summed as a single variable and then compared between cases and controls using the Fisher’s Exact Test (FET) implemented in the R package version R 2.13.1 (http://www.r-project.org). The total number of sequenced chromosomes was counted based on the assumption that each participant had two copies of each chromosome. Because the GC contents of the templates were different, the four *POMC* exons were not amplified with the same success rate. As a result, variable numbers of samples sequenced for each rare variant caused different expected numbers of segregating sites, as shown in previous studies [41]. The harmonic mean approach was thus applied to adjust for the sample size as described by Xie *et al* [42]: 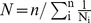, where N_i_ is the sample size of the *i-*th variant and n is the number of variants. The number of each rare variant was adjusted as p_i_*N, where p_i_ is the frequency of *i-*th variant.

### Bioinformatics Analysis

The Transcription Element Search System (TESS, http://www.cbil.upenn.edu/cgi-bin/tess/tess) was used to query for the presence of transcriptional factors (TFs) that could potentially bind to the DNA sequence harboring variants in the 5′ untranslated region (5′ UTR). The program PolyPhen (http://genetics.bwh.harvard.edu/pph) was used to predict the effect of missense variants on protein structure and function. It gives three predictions: benign, possibly damaging, and probably damaging. The multiple sequence alignment program ClusterW, which is incorporated in the BioEdit v7.1.3 software package (http://www.mbio.ncsu.edu/bioedit), was used to identify conserved protein sequences. To predict the function of variants in the 3′ UTR region of *POMC*, miRNAs putatively bound to the sequence containing 3′ UTR variants were identified by the program TargetScanHuman (http://www.targetscan.org/vert_60/). The minimum free energy (MFE) for hybridization of miRNAs to target mRNA sequences was predicted using the program RNAhybrid (http://bibiserv.techfak.uni-bielefeld.de/rnahybrid/). The PhyloP program, which is built into the UCSC genome browser based on multiple alignments of all 46 vertebrate species, was used to calculate the evolutionary conservation score (PhyloP score) of each variant site. The absolute values of the scores represent -log *P*-values under a null hypothesis of neutral evolution. Positive scores indicated that the sites were possibly conserved and negative values indicated that the sites were predicted to be fast-evolving.

## Results

### Identification of *POMC* Exonic Variants

We identified 23 *POMC* exonic variants by direct sequencing, including two common variants and 12 newly discovered rare variants (**Figure 1** **and** **Table 2** **)**. The identified variants have been submitted to the GeneBank (BankIt1533561). One common variant was a 9-bp deletion/insertion polymorphism (c.560_561−/AGCAGCGGC or rs10654394) in the *POMC* coding region (exon 4) and another was a single nucleotide polymorphism (SNP) (c.1130 C>T or rs1042571) in *POMC* 3′ UTR. The frequency of the minor (or 9-bp insertion) allele of marker rs10654394 was 28.4% and 4.8% in AA and EA controls, respectively; the minor allele (T) frequency of SNP rs1042571 was 14.0% and 21.0% in AA and EA controls, respectively. The other 21 variants identified were rare [20 had minor allele frequency (MAF) <1% and one variant (c.61 A>G) had MAF of 0.011 in SD cases] (**Table 2**).

**Table 2:** Common and rare variants identified in *POMC* exons.

ID	Exons	Location	Base change	Amino acid change	Type	Reported in dbSNP	SD cases			Controls		
							AAs	EAs	MAF	AAs	EAs	MAF
							(n/N)	(n/N)		(n/N)	(n/N)	
1	exon1	5'UTR	c.61 A>G			rs139229417	6/270	0/260	0.011	2/208	1/292	0.006
2	exon3	5'UTR	c.259 G>A			No	1/292	0/274	0.002	0/210	0/318	0.000
3	exon3	CDS	c.267 C>T	*P2S*	missense	No	0/292	0/274	0.000	0/210	1/318	0.002
4	exon3	CDS	c.281 C>T	*C6C*	synonymous	rs8192605	0/292	4/274	0.007	0/210	4/318	0.008
5	exon3	CDS	c.343 G>A	*W27stop*	nonsence	No	0/292	0/274	0.000	0/210	1/318	0.002
6	exon4	CDS	c.421 A>G	*D53G*	missense	rs28932470	2/258	0/242	0.004	0/208	1/292	0.002
7	exon4	CDS	c.432 G>T	*E57stop*	nonsence	No	0/258	0/242	0.000	1/208	0/292	0.002
8	exon4	CDS	c.524 C>A	*F87L*	missense	No	2/258	0/242	0.004	0/208	0/292	0.000
9	exon4	CDS	c.545 C>T	*S94S*	synonymous	rs28930368	0/258	4/242	0.008	0/208	2/292	0.004
10	exon4	CDS	c.560_561	*SSG*	3-amino acid	rs10654394	66/258	16/242	0.164	59/208	14/292	0.144
			9-bp Ins	*insertion*	insertion							
11	exon4	CDS	c.609 C>T	*L116L*	synonymous	rs34650613	0/258	0/242	0.000	0/208	1/292	0.002
12	exon4	CDS	c.656 C>A	*G131G*	synonymous	No	1/258	0/242	0.002	0/208	0/292	0.000
13	exon4	CDS	c.685 T>G	*M141R*	missense	No	1/288	0/266	0.002	0/212	0/314	0.000
14	exon4	CDS	c.840 G>T	*G193C*	missense	No	1/288	0/266	0.002	0/212	0/314	0.000
15	exon4	CDS	c.846 G>A	*A195T*	missense	rs141309351	2/288	0/266	0.004	1/212	0/314	0.002
16	exon4	CDS	c.848 C>A	*A195A*	synonymous	rs2071345	0/288	4/266	0.007	0/212	2/314	0.004
17	exon4	CDS	c.867_868	*AG*	2-amino acid	No	0/288	1/266	0.002	0/212	0/314	0.000
			6-bp Ins	insertion	insertion							
18	exon4	CDS	c.904 A>G	*E214G*	missense	rs80326661	0/288	1/266	0.002	0/212	1/314	0.002
19	exon4	CDS	c.969 C>G	*R236G*	missense	rs28932472	0/288	0/266	0.000	0/212	1/314	0.002
20	exon4	CDS	c.989 C>A	*T242T*	synonymous	No	0/288	0/266	0.000	0/212	1/314	0.002
21	exon4	3'UTR	c.1095 T Del			No	0/302	0/282	0.000	0/222	1/324	0.002
22	exon4	3'UTR	c.1130 C>T			rs1042571	35/302	60/282	0.162	31/222	68/324	0.181
23	exon4	3'UTR	c.1187 A>G			No	0/302	0/282	0.000	0/222	1/324	0.002

SD: substance (alcohol, cocaine, opioid and/or marijuana) dependence.

AAs: African Americans; EAs: European Americans.

MAF: minor allele frequency.

n/N: number of rare variants (n)/number of total chromosomes (N).

### Association of Two *POMC* Common Variants with SD or BMI

Genotype distributions of the two common variants (rs10654394 and rs1042571) were consistent with HWE expectations in both AAs and EAs (*P*>0.01, data not shown). As shown in **Table 3**, the common variant rs1042571 in the 3′ UTR was significantly associated with BMI in EAs (Normal weight *vs*. Overweight: *P*_obs_  = 0.003, *P*_emp_  = 0.003, *P*_adj_ = 0.005; Normal weight *vs*. Obese: *P*_obs_  = 0.012, *P*_emp_  = 0.013, *P*_adj_  = 0.018; Normal weight *vs*. Overweight + Obese: *P*_obs_  = 0.001, *P*_emp_  = 0.002, *P*_adj_ = 0.002), but not in AAs. However, there was no association between the common variant rs10654394 and BMI in either AAs or EAs (**Table 3**). The Haploview program [37] was used to determine the phase of the two common SNPs. They were found to be in tight LD (AAs, D′ = 0.87; EAs, D′ = 1.00) but low correlation (AAs, *R^2^* = 0.04; EAs, *R^2^* = 0.02). Haplotype “9 bp Del-T”, consisting of major allele “9 bp Del” of rs10654394 and minor allele T of rs1042571, was significantly associated with BMI in EAs (Normal weight *vs*. Overweight: *P*_obs_  = 0.008, *P*_emp_  = 0.008, *P*_adj_ = 0.011; Normal weight *vs*. Overweight + Obese: *P*_obs_  = 0.016, *P*_emp_  = 0.019, *P*_adj_ = 0.009). Haplotype “ 9 bp Del-C”, consisting of major allele “9 bp Del” of rs10654394 and major allele C of rs1042571, was significantly associated with BMI in EAs (Normal weight *vs*. Overweight: *P*_obs_  = 0.027, *P*_emp_  = 0.033, *P*_adj_ = 0.040; Normal weight *vs*. Overweight + Obese: *P*_obs_  = 0.019, *P*_emp_  = 0.022, *P*_adj_ = 0.016) (**Supporting Information Table S3**). Nevertheless, neither the single maker nor haplotype association analyses revealed association of the two common variants with SD or any specific SD traits in either population (**Supporting Information Tables S4 and S5**).

**Table 3:** Allelic association of two common *POMC* variants and BMI.

Common			Allele	Chi-square tests				Logistic regression analysis		
SNPs	Comparisons	Race	freq*	χ^2^	*P_obs_*	OR (95% CI)	*P_emp_*	STAT	*P_adj_*	OR (95% CI)
rs10654394	Overweight *vs.*	AAs	0.205/0.274	1.90	0.168	0.68 (0.40–1.18)	0.173	−1.38	0.166	0.67 (0.38–1.18)
(9-bp-allele)	Normal weight	EAs	0.043/0.052	0.21	0.646	0.80 (0.32–2.04)	0.691	−0.54	0.586	0.77 (0.30–1.98)
		AAs+EAs	0.119/0.135	0.40	0.526	0.86 (0.55–1.36)	0.557	−1.52	0.130	0.69 (0.43–1.12)
	Obese *vs.*	AAs	0.324/0.274	0.85	0.357	1.27 (0.77–2.10)	0.365	1.06	0.291	1.33 (0.78–2.25)
	Normal weight	EAs	0.081/0.052	1.13	0.289	1.59 (0.67–3.78)	0.310	0.45	0.655	1.23 (0.50–3.03)
		AAs+EAs	0.218/0.135	7.74	0.005	1.79 (1.18–2.71)	0.007	1.24	0.215	1.33 (0.85–2.08)
	Overweight + Obese *vs.*	AAs	0.266/0.274	0.03	0.861	0.96 (0.60–1.52)	0.858	−0.07	0.946	0.98 (0.61–1.58)
	Normal weight	EAs	0.059/0.052	0.09	0.759	1.13 (0.53–2.42)	0.781	−0.03	0.980	0.99 (0.44–2.20)
		AAs+EAs	0.165/0.135	1.57	0.210	1.27 (0.87–1.85)	0.242	−0.08	0.937	0.98 (0.66–1.47)
rs1042571	Overweight *vs.*	AAs	0.141/0.106	0.96	0.328	1.40 (0.72–2.72)	0.372	0.80	0.426	1.30 (0.68–2.49)
(T-allele)	Normal weight	EAs	0.259/0.146	9.11	0.003	2.05 (1.28–3.29)	0.003	2.80	0.005	2.17 (1.26–3.73)
		AAs+EAs	0.202/0.131	7.25	0.007	1.69 (1.15–2.47)	0.011	2.92	0.004	1.81 (1.22–2.69)
	Obese *vs.*	AAs	0.125/0.106	0.29	0.589	1.21 (0.61–2.41)	0.609	0.29	0.769	1.11 (0.54–2.29)
	Normal weight	EAs	0.247/0.146	6.33	0.012	1.92 (1.15–3.20)	0.013	2.37	0.018	2.00 (1.13–3.55)
		AAs+EAs	0.181/0.131	3.38	0.066	1.46 (0.97–2.20)	0.074	2.03	0.043	1.56 (1.02–2.40)
	Overweight + Obese *vs.*	AAs	0.134/0.106	0.73	0.393	1.31 (0.71–2.40)	0.425	0.63	0.529	1.21 (0.66–2.21)
	Normal weight	EAs	0.254/0.146	10.20	0.001	2.00 (1.30–3.06)	0.002	3.06	0.002	2.13 (1.31–3.45)
		AAs+EAs	0.193/0.131	6.75	0.009	1.58 (1.12–2.24)	0.011	2.91	0.004	1.71 (1.19–2.46)

rs10654394: a 9-bp insertion/deletion polymorphism (−/AGCAGCGGC) in *POMC* exon 4; rs1042571: C/T in *POMC* 3′UTR.

AAs: African Americans; EAs: European Americans.

Normal weight: BMI  = 18.5–24.9; Overweight: BMI  = 25–29.9; Obese: BMI ≥30.

*P*_obs_: observed *P* values calculated by Chi-square tests; *P*_emp_: empirical *P* values using 10,000 permutations; *P*_adj_: *P* values obtained from multivariate logistic regression analysis and adjusted by sex, age, substance dependence (SD) status, and ancestry proportion.

Allele freq*: numbers before the slash symbol “/” represent the allele frequency in the conditioned group (i.e., overweight, obese, or over weight + obese groups), and numbers after the slash symbol “/” represent the allele frequency in the comparison group (i.e., the normal weight group).

### Compound Effects of Rare *POMC* Exonic Variants on SD or BMI

**Figure 2:**
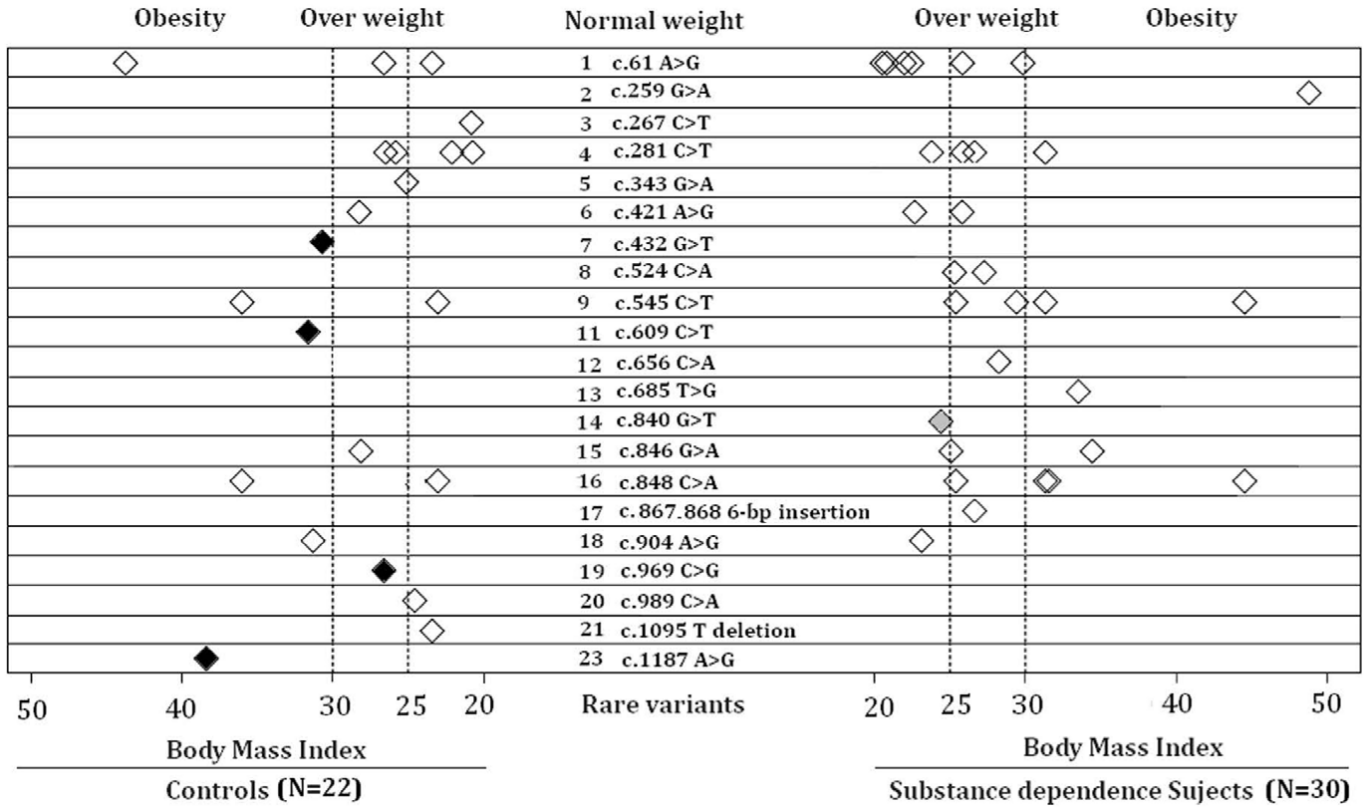
Scatter plots of subjects with rare variants according to substance dependence (SD) and BMI. Each rhombus represents a subject with a rare variant. Each row across the Y axis represents one type of rare variants. The X-axis represents two major groups: cases with substance dependence (SD) (on right side) and controls (on left side). Within each group, subjects carrying rare variants are divided into three groups according to BMI scores: the normal-weight group (BMI: 18.5–24.9), the over-weight group (BMI: 25–29.9), and the obese group (BMI ≥30). Overweight control subjects are represented with dark rhombuses and normal weight SD cases are represented with grey rhombuses.

Fisher’s exact tests with 21 rare variants collapsed as a single variable were performed and the results of data analysis are summarized in **Table 4**. In AAs, rare *POMC* exonic variants were significantly more frequent in SD cases overall or in cases with AD, CD or MjD specifically than in controls (SD: *P*_FET, 1df_  = 0.026; AD: *P*_FET, 1df_  = 0.027; CD: *P*_FET, 1df_  = 0.007; MjD: *P*_FET, 1df_  = 0.050). However, in EAs, the frequency of rare *POMC* exonic variants did not differ significantly between SD cases and controls (**Table 4**). Bonferroni correction was used to adjust the empirical *P* values obtained from the Fisher Exact test, resulting in a significance level *P*_correction_ <0.01 (0.05/5, i.e., correction for five times of comparisons in each sample set). Only the *P* value (*P*_FET, 1df_  = 0.007) obtained from the analysis of CD data in AAs survived Bonferroni correction. In addition, rare *POMC* exonic variants did not occur significantly more frequently in the overweight or obese group than in the normal weight group in either population (**Table 4**). Nevertheless, we observed that certain exonic variants (c.432 G>T, c.609 C>T, c.969 C>G and c.1187 A>G) appeared only in overweight or obese subjects unaffected with SD (**Figure 2**).

**Table 4:** Collapse association of *POMC* rare variants with substance dependence (SD) or body mass index (BMI).

	African Americans (AAs)		European Americans (EAs)		AAs + EAs	
	Counts	*P*_FET_,_1df_	Counts	*P*_FET_,_1df_	Counts	*P*_FET_,_1df_
All SD cases *vs*. Controls	16/265, 4/208	0.026	14/248, 18/290	0.471	30/512, 22/498	0.200
AD cases *vs*. Controls	12/209, 3/195	0.027	12/194, 17/288	0.525	24/402, 20/483	0.151
CD cases *vs*. Controls	13/191, 3/210	0.007	11/143, 18/286	0.374	24/334, 21/498	0.056
OD cases *vs*. Controls	3/36, 4/208	0.078	6/78, 18/290	0.411	9/115, 22/498	0.121
MjD cases *vs*. Controls	7/109, 4/208	0.050	7/75, 18/290	0.256	14/184, 22/498	0.088
Normal weight *vs.* Overweight	6/128, 9/172	0.530	10/216, 11/188	0.381	16/344, 20/360	0.365
Normal weight *vs.* Obese	6/128, 5/172	0.316	10/216, 11/135	0.149	16/344, 16/307	0.446
Normal weight *vs.* Overweight + Obese	6/128, 14/344	0.475	10/216, 22/323	0.212	16/344, 36/667	0.374

SD: substance (alcohol, cocaine, opioid and/or marijuana) dependence.

AD: alcohol dependence; CD: cocaine dependence; OD: opioid dependence; MjD: marijuana dependence.

Normal weight: BMI  = 18.5–24.9; Overweight: BMI  = 25–29.9; Obese: BMI ≥30;

*P*_FET,_ _1df_: *P* values obtained using the rare variant collapsing method and the Fisher Exact test (df  = 1).

Counts: adjusted numbers of minor (before the slash symbol "/") and total (after the slash symbol "/") alleles in the conditioned group (i.e., subjects with SD or high BMI, on the left side) and the comparison group (i.e., control subjects or subjects with normal BMI, on the right side) using the harmonic mean method [42].

### Functional Prediction of *POMC* Exonic Variants

The predicted function of *POMC* exonic variants is presented in **Table 5**. The two rare variants (c.61 A>G and c.259 G>A) in the 5′ UTR of *POMC* were predicted to be located in transcription factor binding sites. Variant c.61 A>G was observed in nine heterozygous individuals. The variant allele G was predicted to be located in the binding site of transcription factor E2F, and the common allele A was potentially located in the binding site of transcription factors Sp1 and CAC-binding protein. Variant c.259 G>A was found in only one heterozygous carrier. The variant allele A was predicted to be located in the binding site of transcription factor GR, and the common allele G was potentially located in the binding site of transcription factor myogenin.

**Table 5:** Functional prediction of identified *POMC* rare variants.

Location	ID	Variants	PhyloP score	Function prediction
5' UTR	1	c.61 A>G	−1.16	*E2F* (allele G); Sp1 and CAC binding protein (allele A)
	2	c.259 G>A	0.36	*GR* (allele A); myogenin (allele G)
Coding	3	c.267 C>T	2.73	*P2S*, predicted to be probably damaging
regions	4	c.281 C>T	0.28	*C6C*, synonymous
	5	c.343 G>A	1.32	*W27Stop*, nonsence
	6	c.421 A>G	0.30	*D53G*, predicted to be benign
	7	c.432 G>T	2.58	*E57stop*, nonsence
	8	c.524 C>A	−0.49	*F87L*, predicted to be probably damaging
	9	c.545 C>T	0.50	*S94S*, synonymous
	10	c.560_561 9-bp ins	0.99	Three amino acid (SSG) insertion
	11	c.609 C>T	−0.05	*L116L*, synonymous
	12	c.656 C>A	0.10	*G131G*, synonymous
	13	c.685 T>G	1.93	*M141R*, predicted to be probably damaging
	14	c.840 G>T	−0.47	*G193C*, predicted to be probably damaging
	15	c.846 G>A	0.00	*A195T*, predicted to be probably damaging
	16	c.848 C>A	0.35	*A195A*, synonymous
	17	c.867_868 6-bp ins	−3.71	Two amino acid (AG) insertion
	18	c.904 A>G	−0.34	*E214G*, predicted to be possibility damaging
	19	c.969 C>G	2.38	*R236G*, predicted to be probably damaging
	20	c.989 C>A	0.50	*T242T*, synonymous
3' UTR	21	c.1095 T del	0.03	hsa-mir-4728-5p (−26.2 kcal/mol > −23.4 kcal/mol)
				hsa-mir-4723-5p (−22.8 kcal/mol > −23.8 kcal/mol)
				hsa-mir-4525 (−25.6 kcal/mol > −27.2 kcal/mol)
				hsa-mir-4665-5p (−27.4 kcal/mol > −24.0 kcal/mol)
				hsa-mir-1275 (−26.5 kcal/mol > −22.2 kcal/mol)
				hsa-mir-625 (−23.4 kcal/mol > −21.5 kcal/mol)
	22	c.1130 G>T	−0.66	hsa-mir-3715 (−25.0 kcal/mol > −22.1 kcal/mol)
				hsa-mir-1909 (−26.7 kcal/mol > −24.9 kcal/mol)
	23	c.1187 A>G	−0.02	No miRNAs were predicted

5′ UTR: transcription factors (TFs) were queried using DNA sequences harboring variants in the 5′ untranslated regions (5' UTRs) against the Transcription Element Search System.

Coding Region: potential functional and structural changes caused by missense variants in coding regions were predicted by the program PolyPhen.

3' UTR: microRNAs targeting 3′ untranslated region (3' UTR) sequences containing variants were predicted by program TargetScan, and changes in minimal free energy (mfe) due to mutations are presented in parentheses.

PhyloP score or evolutionary conservation score: calculated by the program PhyloP, which is built into the UCSC genome browser based on multiple alignments of 46 vertebrate species.

In the coding region, two variants (c.343 G>A and c.432 G>T) were predicted to be nonsense mutations that potentially terminate *POMC* mRNA translation. Each of them was identified in only one heterozygous carrier. Variant c.867_868 6-bp (GGGCCC) caused a two-amino acid (Ala-Gly) insertion. This insertion variant is located in the β-LPH domain. Seven missense mutations (c.267 C>T, c.524 C>A, c.685 T>G, c.840 G>T, c.846 G>A, c.904 A>G, and c.969 C>G) were predicted to be damaging and one missense mutations (c.421 A>G) were predicted to be benign. Levels of evolutionary conservation of rare variants in coding regions were described at both the nucleotide and amino acid levels. Comparing the *POMC* coding sequence of humans with those of 45 other vertebrate species showed that most of the identified rare variants had a positive PhyloP score, indicating that they tended to be conservative rather than fast-evolving (**Table 5**). Additionally, alignment of POMC amino acid sequence across multiple species showed that the rare variants in coding regions were located in highly conserved regions (**Supporting Information Figure S1**).

In the 3′UTR of *POMC*, variant c.1095 T del was predicted to be located in the target site of six miRNAs. The variant deletion allele may cause an increased MFE for four miRNAs (hsa-mir-4728-5p, hsa-mir-4665-5p, hsa-mir-1275 and hsa-mir-625) but a decreased MFE for two miRNAs (hsa-mir-4723-5p and hsa-mir-4525). Variant c.1130 G>T was predicted to be located in the target site of two miRNAs (hsa-mir-3715 and hsa-mir-1909) and the variant allele T may result in an increased MFE for these two miRNAs. Variant c.1187 A>G was not predicted to be located in the target site of any miRNAs (**Table 5**).

## Discussion

Melanocortin peptides such as ACTH, MSH and β-endorphin are derived from the precursor molecule POMC, which is encoded by the POMC gene (*POMC*). Variation in *POMC* may contribute to SD [20], [21], [22], [23] and obesity [24], [26]. The present study focused on the identification of *POMC* exonic variants and the association of *POMC* exonic variants with SD (specifically, AD, CD, OD and/or MjD) and BMI. Our findings suggest that *POMC* exonic variants may influence risk for both SD and BMI. Nevertheless, *POMC* common and rare variants may exert different effects on these two phenotypes.

In this study, two common *POMC* exonic variants [the 9-bp insertion/deletion polymorphism (c.560_561 9-bp Ins or rs10654394) in exon 4 and c.1130 G>T (or rs1042571) in the 3′ UTR] were identified and their association with SD or BMI was analyzed. A positive association between common variant rs1042571 and BMI was observed in EAs but not AAs. In a previous study, the common variant rs10654394 (or the 9-bp insertion/deletion polymorphism) was associated with obesity in children [26]. However, in the present study, we saw no association between this variant and BMI in either population. Additionally, the two common variants were not associated with SD in either AAs or EAs. In fact, no published studies have demonstrated an association between these two common exonic variants and SD. To understand whether our sample had sufficient statistical power to detect the association between these two common variants and SD or BMI, a retrospective power analysis was conducted. Assuming the statistical power equals to 80% and the type I error equals to 0.05, the minimum effect size (or odds ratio) that is detectable would be 1.67 (in AAs) and 2.50 (in EAs) for variant rs10654394 and 2.00 (in AAs) and 1.68 (in EAs) for variant rs1042571. Considering that the sample size for this study was moderate, further studies with a larger sample are needed to validate the above findings.

Moreover, the two common variants are potentially functional. The 9-bp insertion allele of rs10654394 (c.560_561 9-bp Ins) leads to three extra amino acids (Ser-Ser-Gly) at the carboxyl terminus of γ-MSH in the conserved region of the 16 KD fragment (**Figure 1**). This variant may influence mRNA stability or post-translational cleavage of the POMC peptide. The variant rs1042571 (c.1130 G>T) in the 3′ UTR was predicted to be located in the binding site of two miRNAs (hsa-mir-3715 and hsa-mir-1909) (**Table 5**). The variant allele T potentially increases the minimal free energy (MFE) for hybridization of these two miRNAs (hsa-mir-3715: from −25.0 kcal/mol to −22.1 kcal/mol; hsa-mir-1909: from −26.7 kcal/mol to −24.9 kcal/mol) to the target sequence in *POMC* 3′ UTR, thus reducing the binding of miRNAs to *POMC* 3′ UTR and increasing *POMC* expression.

Additionally, we identified 21 rare variants in *POMC* exons, most of which were predicted to cause a functional or structural change in POMC as described in **Table 5**. When these exonic variants were collapsed into a single variable and the frequency of which was compared between SD cases and controls, a significantly higher frequency of these rare variants was observed in AA cases affected with SD in general or AD, CD or MjD specifically than in AA controls. Nevertheless, in EAs, these rare variants were not significantly more frequent in SD cases than controls (**Table 4**). Thus, rare *POMC* exonic variants may contribute to the etiology of SD in a population-specific pattern. Moreover, the association of these rare variants with overweight or obesity was analyzed using the same approach but no positive findings were obtained (**Table 4**). In this study, 21 rare variants were included in Fisher’s exact tests. Because they were collapsed into a single variable to test their cumulative influence on SD or BMI, corrections for multiple testing (e.g., Bonferroni correction) were not applied to adjust the significance *P* values.

Although *POMC* rare variants were not found to be associated with BMI using the collapsing method and the Fisher’s exact test, we cannot exclude the possibility that certain rare *POMC* exonic variants may influence overweight or obesity. As shown in **Figure 2**, eight rare variants (c.267 C>T, c.343 G>A, c.432 G>T, c.609 C>T, c.969 C>G, c.989 C>A, c.1095 T deletion, and c.1187 A>G) were identified only in controls, and each of them was identified in a single heterozygous carrier. Four of them (c.432 G>T, c.609 C>T, c.969 C>G and c.1187 A>G) were only present in control subjects with BMI >25.0, suggesting that these rare variants may increase risk of becoming overweight or obese specifically. In addition, rare *POMC* exonic variants may confer risk for SD and overweight or obesity through a shared mechanism. As shown in **Figure 2**, five rare variants (c.259 G>A, c.524 C>A, c.656 C>A, c.685 T>G, and c.867_868 6-bp Ins) were identified only in SD cases who had overweight or obesity. Thus, these rare *POMC* exonic variants may influence the vulnerability to SD and overweight or obesity via a common biological pathway.

As we know, exonic rare variants may have a larger impact on gene transcription or protein activity than intronic or intergenic variants, and in some cases, they may cause the disease directly. Hence, it would be important to explore the biological function of the rare variants or mutations in *POMC* exons. As shown in **Table 5**, *POMC* exonic variants may change the affinity of transcription factors for their binding sites (e.g., variants in the 5′ UTR), introduce premature stop codons (e.g., nonsense mutations in coding regions), alter amino acid sequences (e.g., missense mutations in coding regions), or change the activity of regulatory miRNAs (e.g., variants in the 3′ UTR).

The findings from this study should be seen in the context of three main limitations: (1) a moderate sample size, (2) the function of exonic variants was predicted only by bioinformatics analyses, and (3) the population stratification issue was not considered in rare variant data analysis. A larger sample would increase the potential to identify *POMC* rare variants and provide a greater statistical power to analyze the association of identified variants and diseases. This is particularly important for detection of small or moderate effects of genes involved in complex disorders. Because the etiological role of the rare variants identified in this study was only predicted using bioinformatics analysis, experimental studies *in vivo* or *in vitro* are needed to validate their biological function. Another challenge was to control the influence of potential population substructure bias on association analysis results. Matheson and McVean [40] pointed out that population structure could alter the conclusion of genetic association studies involving either common or rare variants. Principle component analysis is the usual method used to control for population structure in genome-wide association studies, in which the top components are considered as covariates. In this study, we used the ancestry coefficients obtained from running STRUCTURE [32], [33] on 41 ancestry informative markers to exclude participants whose genetic ancestry conflicted with their self-reported race and took the score as a continuous covariate to adjust for the genetic background noise for the two identified common variants. However, there is no accepted method to correct the effect of population stratification on rare variant analysis results per our current design. One possible way, as suggested by Matheson and McVean [40], is that family-based association tests, by inclusion of other family members of participants, might help minimize the influence of structured populations on association analysis results.

In conclusion, this study provides evidence that both common and rare variants in *POMC* could increase the risk for SD and/or obesity. It also suggests that certain *POMC* variants may influence the vulnerability to SD and overweight or obesity in a common or specific biological pathway. Functional studies are needed to elucidate the shared or specific molecular mechanisms by which *POMC* variation influences the susceptibility to SD and/or obesity.

## Supporting Information

Figure S1 **Conserved analysis of POMC protein sequence across multiple species.**
(TIF)

Table S1 **Primers for sequencing four** ***POMC*** **exons.**
(DOC)

Table S2 **PCR conditions for amplifying four** ***POMC*** **exons.**
(DOC)

Table S3 **Haplotype association of two common** ***POMC*** **variants (rs1654394-rs1042571) and BMI.**
(DOC)

Table S4 **Allelic association of two** ***POMC*** **common variants and substance dependence (SD) traits.**
(DOC)

Table S5 **Haplotype association of two** ***POMC*** **common variants (rs10654394-rs1042571) and substance dependence (SD).**
(DOC)
