# Supplementary figures and images for: Identification of POMC Exonic Variants Associated with Substance Dependence and Body Mass Index
Source: PLoS One. 2012 Sep 17;7(9):e45300. doi: 10.1371/journal.pone.0045300 (PMC3444488; doi:10.1371/journal.pone.0045300)

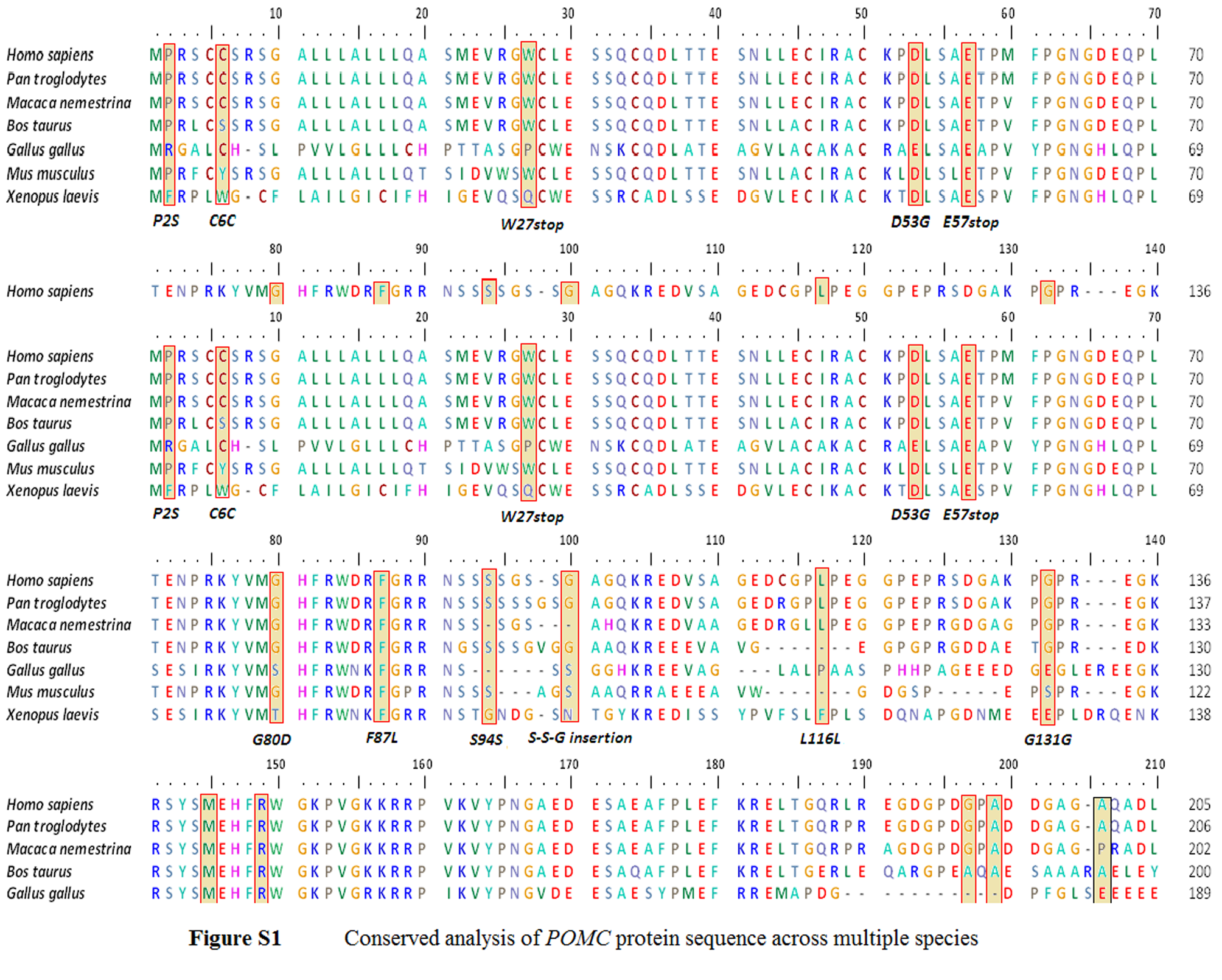

Supplement: Figure S1 — Conserved analysis of POMC protein sequence across multiple species. (TIF) [file pone.0045300.s001.tif]
