# Supplementary material for: Identification of POMC Exonic Variants Associated with Substance Dependence and Body Mass Index
Source: PLoS One. 2012 Sep 17;7(9):e45300. doi: 10.1371/journal.pone.0045300 (PMC3444488; doi:10.1371/journal.pone.0045300)
Supplement: Table S1 — Primers for sequencing four POMC exons. (DOC) [file pone.0045300.s002.doc]

| **Table S1.** Primers for sequencing four *POMC* exons. | | | |
| --- | --- | --- | --- |
|  |  | **Forward primers (5'-3')** | **Reverse primers (5'-3')** |
| PCR primers | Exon 1 | gttctaagcggagacccaac | gcgcagaaagtttgtcgag |
|  | Exon 2 | gttggcggtgagctgatct | tctggctcatcttctggaca |
|  | Exon 3 | accgctgtactgcacacctc | agctgcttccttccctgatt |
|  | Exon 4 | gtgggcagacctgctctgta | tatcctaccgcatggaaacc |
|  |  |  |  |
| Sequencing primers | Exon 1 | gttcttcctgagggcgagcg | gacggggacaggggatc |
|  | Exon 2 | cccacctggttaagggaaat | atggcaggatttgaagaggatg |
|  | Exon 3 | ccttaaaatgcctttaactcc | cacgcctatcactgggaatg |
|  | Exon 4-1 | ggcacgtggcgagggcggcc | cagctccctcttgaactccag |
|  | Exon 4-2 | gacctccgagaagagccaga | tgccctcactcgcccttc |
|  | Exon 4-3 | cgcccagtgaaggtgtacc | atccatgctgctgttatttgac |

Primers were designed according to genomic sequence from the Ensembl database (*POMC*, ENST00000380794).
