# Supplementary material for: Identification of POMC Exonic Variants Associated with Substance Dependence and Body Mass Index
Source: PLoS One. 2012 Sep 17;7(9):e45300. doi: 10.1371/journal.pone.0045300 (PMC3444488; doi:10.1371/journal.pone.0045300)
Supplement: Table S2 — PCR conditions for amplifying four POMC exons. (DOC) [file pone.0045300.s003.doc]

| **Table S2.** PCR conditions for amplifying four *POMC* exons. | | | | |
| --- | --- | --- | --- | --- |
| **PCR Reaction Mix** | **Exon 1^a^** | **Exon 2^~~a~~^** | **Exon 3^a^** | **Exon 4^b^** |
| PCR reaction buffer | 2 | 2 | 2 | 6 |
| 10 mM dNTP mixture | 0.4 | 0.4 | 0.4 | 0.6 |
| 50 mM MgCl_2_ | 0.4 | 0.8 | 0.8 | 0 |
| DMSO | 0 | 0 | 0 | 0.9 |
| Forward primer (10 μM) | 1 | 1 | 1 | 1.5 |
| Reverse primer (10 μM) | 1 | 1 | 1 | 1.5 |
| ddH_2_O | 13.15 | 12.75 | 12.75 | 17.4 |
| Genomic DNA (10 ng/μl) | 2 | 2 | 2 | 2 |
| Taq DNA Polymerase | 0.05 | 0.05 | 0.02 | 0.1 |
| Total volume | 20 (μL) | 20(μL) | 20(μL) | 30(μL) |
| PCR programs |  |  |  |  |
| Step 1 (1 cycle) |  |  |  |  |
| Denature (3min) | 94°C | 94°C | 94°C | 94°C |
| Step 2 (20 cycles) |  |  |  |  |
| Denature (30 sec) | 94°C | 94°C | 94°C | 94°C |
| Anneal (touchdown) (30 sec) | 65-50°C | 65-55°C | 67-55°C | 69-57°C |
| Extend (30 sec) | 72°C | 72°C | 72°C | 72°C |
| Step 3 (20 cycles) |  |  |  |  |
| Denature (30 sec) | 94°C | 94°C | 94°C | 94°C |
| Anneal (30 sec) | 50°C | 55°C | 55°C | 57°C |
| Extention (30 sec) | 72°C | 72°C | 72°C | 72°C |
| Step 4 (1 cycle) |  |  |  |  |
| Extent (10 min) | 72°C | 72°C | 72°C | 72°C |

^a^10× PCR buffer minus Mg^2+^ and Taq DNA Polymerase were purchased from Life Technologies, Grand Island, NY, USA.

^b^5× Phusion HF Buffer and High-Fidelity DNA polymerase were purchased from New England Biolabs, Ipswich, MA, USA.
