# Supplementary material for: Identification of POMC Exonic Variants Associated with Substance Dependence and Body Mass Index
Source: PLoS One. 2012 Sep 17;7(9):e45300. doi: 10.1371/journal.pone.0045300 (PMC3444488; doi:10.1371/journal.pone.0045300)
Supplement: Table S3 — Haplotype association of two common POMC variants (rs1654394-rs1042571) and BMI. (DOC) [file pone.0045300.s004.doc]

**Table S3.** Haplotype association of two common *POMC* variants (rs1654394-rs1042571) and BMI

| Haplotypes | Type | Population | Allele Freq* | χ2 | *P_obs_* | *P*_emp_ | STAT | *P_adj_* | OR |
| --- | --- | --- | --- | --- | --- | --- | --- | --- | --- |
| 9bp Del-T | model 1 | AAs | 0.136/0.098 | 0.934 | 0.334 | 0.367 | 0.84 | 0.360 | 1.43 |
| 9bp Del-T | Model 1 | EAs | 0.255/0.149 | 6.985 | **0.008** | **0.008** | 6.52 | **0.011** | 2.17 |
| 9bp Del-T | Model 1 | AAs+EAs | 0.201/0.131 | 5.963 | **0.015** | **0.016** | 7.29 | **0.007** | 1.84 |
| 9bp Del-T | Model 2 | AAs | 0.117/0.098 | 0.253 | 0.615 | 0.621 | 0.10 | 0.747 | 1.14 |
| 9bp Del-T | Model 2 | EAs | 0.206/0.149 | 1.870 | 0.172 | 0.174 | 3.41 | 0.065 | 1.84 |
| 9bp Del-T | Model 2 | AAs+EAs | 0.157/0.131 | 0.884 | 0.347 | 0.342 | 2.09 | 0.148 | 1.43 |
| 9bp Del-T | Model 3 | AAs | 0.126/0.098 | 0.662 | 0.416 | 0.433 | 0.49 | 0.484 | 1.28 |
| 9bp Del-T | Model 3 | EAs | 0.235/0.149 | 5.767 | **0.016** | **0.019** | 6.92 | **0.009** | 2.04 |
| 9bp Del-T | Model 3 | AAs+EAs | 0.180/0.131 | 3.922 | **0.048** | 0.053 | 6.10 | **0.014** | 1.66 |
|  |  |  |  |  |  |  |  |  |  |
| 9bp Ins-C | Model 1 | AAs | 0.206/0.282 | 2.189 | 0.139 | 0.135 | 2.35 | 0.126 | 0.64 |
| 9bp Ins-C | Model 1 | EAs | 0.043/0.053 | 0.231 | 0.631 | 0.651 | 0.33 | 0.565 | 0.76 |
| 9bp Ins-C | Model 1 | AAs+EAs | 0.117/0.137 | 0.587 | 0.444 | 0.465 | 2.87 | 0.090 | 0.66 |
| 9bp Ins-C | Model 2 | AAs | 0.338/0.282 | 1.010 | 0.315 | 0.310 | 1.11 | 0.292 | 1.33 |
| 9bp Ins-C | Model 2 | EAs | 0.081/0.053 | 1.077 | 0.299 | 0.310 | 0.17 | 0.682 | 1.21 |
| 9bp Ins-C | Model 2 | AAs+EAs | 0.223/0.137 | 7.964 | **0.005** | **0.007** | 1.47 | 0.226 | 1.32 |
| 9bp Ins-C | Model 3 | AAs | 0.274/0.282 | 0.031 | 0.860 | 0.844 | 0.02 | 0.898 | 0.97 |
| 9bp Ins-C | Model 3 | EAs | 0.059/0.053 | 0.079 | 0.779 | 0.798 | 0.01 | 0.950 | 0.98 |
| 9bp Ins-C | Model 3 | AAs+EAs | 0.167/0.137 | 1.455 | 0.228 | 0.253 | 0.03 | 0.857 | 0.96 |
|  |  |  |  |  |  |  |  |  |  |
| 9bp Del-C | Model 1 | AAs | 0.658/0.620 | 0.436 | 0.509 | 0.576 | 0.37 | 0.545 | 1.16 |
| 9bp Del-C | Model 1 | EAs | 0.702/0.798 | 4.882 | **0.027** | **0.033** | 4.22 | **0.040** | 0.57 |
| 9bp Del-C | Model 1 | AAs+EAs | 0.682/0.733 | 2.071 | 0.150 | 0.139 | 1.25 | 0.264 | 0.82 |
| 9bp Del-C | Model 2 | AAs | 0.545/0.620 | 1.591 | 0.207 | 0.187 | 1.54 | 0.214 | 0.72 |
| 9bp Del-C | Model 2 | EAs | 0.713/0.798 | 3.288 | 0.070 | 0.061 | 3.60 | 0.058 | 0.57 |
| 9bp Del-C | Model 2 | AAs+EAs | 0.620/0.733 | 9.106 | **0.003** | **0.002** | 4.51 | **0.034** | 0.67 |
| 9bp Del-C | Model 3 | AAs | 0.600/0.620 | 0.142 | 0.706 | 0.674 | 0.17 | 0.677 | 0.91 |
| 9bp Del-C | Model 3 | EAs | 0.707/0.798 | 5.521 | **0.019** | **0.022** | 5.84 | **0.016** | 0.55 |
| 9bp Del-C | Model 3 | AAs+EAs | 0.653/0.733 | 6.301 | **0.012** | **0.011** | 3.84 | **0.050** | 0.73 |

rs10654394: a 9-bp insertion/deletion polymorphism (-/AGCAGCGGC) in *POMC* exon 4; rs1042571: a SNP marker (C/T) in *POMC* 3’UTR.

AAs: African Americans; EAs: European Americans.

*P*_obs_: observed *P* values calculated by Chi-square tests.

*P*_emp_: empirical *P* values using 10,000 permutations.

*P_adj_*, *P* values adjusted by sex, age, substance dependence status, and race using multivariate logistic regression analysis.

Model 1: over-weight *vs*. normal weight.

Model 2: obese *vs*. normal weight.

Model 3: over-weight + obese *vs*. normal weight.

Allele Freq*: numbers before the slash symbol “/” represent the allele frequency in the conditioned group (Overweight group, Obese, or Over weight + Obese), and numbers after the slash symbol “/” represent the allele frequency in the comparison group (Normal weight).
