# Supplementary material for: Identification of POMC Exonic Variants Associated with Substance Dependence and Body Mass Index
Source: PLoS One. 2012 Sep 17;7(9):e45300. doi: 10.1371/journal.pone.0045300 (PMC3444488; doi:10.1371/journal.pone.0045300)
Supplement: Table S4 — Allelic association of two POMC common variants and substance dependence (SD) traits. (DOC) [file pone.0045300.s005.doc]

**Table S4.** Allelic association of two *POMC* common variants and substance dependence (SD) traits.

| Comparisons | Race | Allele freq. | Chi-square tests | | | *P_emp_* | Logistic regression analysis | | |
| --- | --- | --- | --- | --- | --- | --- | --- | --- | --- |
|  |  | (Case/Control) | χ^2^ | *P_obs_* | OR (95% CI) |  | STAT | *P_adj_* | OR (95% CI) |
| ***rs10654394 (9-bp-allele)*** |  |  |  |  |  |  |  |  |  |
| SD cases *vs*. Controls | AAs | 0.256/0.284 | 0.46 | 0.5 | 0.87 (0.58-1.31) | 0.493 | -0.97 | 0.334 | 0.81 (0.53-1.24) |
| AD cases *vs*. Controls | AAs | 0.238/0.286 | 1.19 | 0.275 | 0.78 (0.50-1.22) | 0.275 | -1.22 | 0.223 | 0.75 (0.46-1.20) |
| CD cases *vs*. Controls | AAs | 0.280/0.289 | 0.04 | 0.845 | 0.96 (0.61-1.49) | 0.848 | -0.29 | 0.772 | 0.93 (0.59-1.48) |
| OD cases *vs*. Controls | AAs | 0.344/0.284 | 0.49 | 0.486 | 1.32 (0.60-2.91) | 0.492 | 0.57 | 0.569 | 1.26 (0.56-2.83) |
| MjD cases *vs*. Controls | AAs | 0.206/0.284 | 2.16 | 0.142 | 0.65 (0.37-1.15) | 0.145 | -1.52 | 0.129 | 0.64 (0.35-1.14) |
| SD cases *vs*. Controls | EAs | 0.066/0.048 | 0.82 | 0.364 | 1.41 (0.67-2.94) | 0.384 | 0.84 | 0.404 | 1.38 (0.65-2.95) |
| AD cases *vs*. Controls | EAs | 0.072/0.048 | 1.22 | 0.27 | 1.53 (0.71-3.29) | 0.296 | 0.97 | 0.333 | 1.48 (0.67-3.26) |
| CD cases *vs*. Controls | EAs | 0.082/0.048 | 1.94 | 0.164 | 1.78 (0.78-4.02) | 0.188 | 1.19 | 0.235 | 1.67 (0.72-3.90) |
| OD cases *vs*. Controls | EAs | 0.071/0.048 | 0.63 | 0.429 | 1.53 (0.53-4.39) | 0.451 | 1.01 | 0.313 | 1.72 (0.60-4.90) |
| MjD cases *vs*. Controls | EAs | 0.053/0.048 | 0.03 | 0.866 | 1.10 (0.35-3.45) | 0.873 | 0.05 | 0.964 | 1.03 (0.31-3.44) |
| SD cases *vs*. Controls | AAs+EAs | 0.164/0.146 | 0.62 | 0.432 | 1.15 (0.81-1.62) | 0.464 | -0.73 | 0.463 | 0.87 (0.60-1.26) |
| AD cases *vs*. Controls | AAs+EAs | 0.157/0.144 | 0.27 | 0.604 | 1.10 (0.76-1.60) | 0.618 | -0.79 | 0.428 | 0.85 (0.56-1.28) |
| CD cases *vs*. Controls | AAs+EAs | 0.196/0.147 | 3.35 | 0.067 | 1.41 (0.97-2.05) | 0.083 | 0.00 | 0.999 | 1.00 (0.67-1.50) |
| OD cases *vs*. Controls | AAs+EAs | 0.157/0.146 | 0.08 | 0.778 | 1.09 (0.60-1.96) | 0.787 | 0.78 | 0.435 | 1.29 (0.68-2.43) |
| MjD cases *vs*. Controls | AAs+EAs | 0.140/0.146 | 0.03 | 0.857 | 0.96 (0.59-1.56) | 0.862 | -1.6 | 0.11 | 0.64 (0.37-1.11) |
|  |  |  |  |  |  |  |  |  |  |
| ***rs1042571 (T-allele)*** |  |  |  |  |  |  |  |  |  |
| SD cases *vs*. Controls | AAs | 0.116/0.140 | 0.66 | 0.418 | 0.81 (0.48-1.36) | 0.443 | -0.43 | 0.669 | 0.89 (0.54-1.49) |
| AD cases *vs*. Controls | AAs | 0.100/0.139 | 1.66 | 0.198 | 0.69 (0.39-1.22) | 0.213 | -0.77 | 0.444 | 0.80 (0.46-1.40) |
| CD cases *vs*. Controls | AAs | 0.122/0.142 | 0.4 | 0.525 | 0.83 (0.48-1.46) | 0.542 | -0.3 | 0.764 | 0.92 (0.53-1.59) |
| OD cases *vs*. Controls | AAs | 0.146/0.140 | 0.01 | 0.911 | 1.05 (0.43-2.55) | 0.913 | 0.65 | 0.515 | 1.32 (0.57-3.03) |
| MjD cases *vs*. Controls | AAs | 0.119/0.140 | 0.3 | 0.586 | 0.83 (0.43-1.61) | 0.609 | -0.06 | 0.952 | 0.98 (0.50-1.91) |
| SD cases *vs*. Controls | EAs | 0.213/0.210 | 0.01 | 0.931 | 1.02 (0.69-1.50) | 0.949 | -0.14 | 0.889 | 0.97 (0.64-1.47) |
| AD cases *vs*. Controls | EAs | 0.200/0.214 | 0.15 | 0.698 | 0.92 (0.60-1.41) | 0.71 | -0.74 | 0.460 | 0.84 (0.53-1.33) |
| CD cases *vs*. Controls | EAs | 0.223/0.210 | 0.11 | 0.74 | 1.08 (0.69-1.70) | 0.743 | -0.01 | 0.990 | 1.00 (0.62-1.61) |
| OD cases *vs*. Controls | EAs | 0.245/0.210 | 0.54 | 0.462 | 1.22 (0.72-2.08) | 0.47 | 0.35 | 0.726 | 1.11 (0.62-1.98) |
| MjD cases *vs*. Controls | EAs | 0.159/0.210 | 1.12 | 0.29 | 0.71 (0.38-1.34) | 0.302 | -1.14 | 0.253 | 0.65 (0.31-1.36) |
| SD cases *vs*. Controls | AAs+EAs | 0.163/0.181 | 0.69 | 0.406 | 0.88 (0.64-1.20) | 0.435 | -0.34 | 0.731 | 0.95 (0.69-1.30) |
| AD cases *vs*. Controls | AAs+EAs | 0.148/0.184 | 2.36 | 0.125 | 0.77 (0.55-1.08) | 0.135 | -0.90 | 0.369 | 0.85 (0.60-1.21) |
| CD cases *vs*. Controls | AAs+EAs | 0.166/0.183 | 0.44 | 0.508 | 0.89 (0.63-1.26) | 0.526 | -0.23 | 0.820 | 0.96 (0.67-1.37) |
| OD cases *vs*. Controls | AAs+EAs | 0.212/0.181 | 0.73 | 0.394 | 1.22 (0.77-1.91) | 0.421 | 0.57 | 0.569 | 1.14 (0.72-1.82) |
| MjD cases *vs*. Controls | AAs+EAs | 0.136/0.181 | 2.3 | 0.129 | 0.71 (0.45-1.11) | 0.145 | -0.75 | 0.454 | 0.83 (0.52-1.34) |

rs10654394: a 9-bp insertion/deletion polymorphism (-/AGCAGCGGC) in *POMC* exon 4; rs1042571: a SNP marker (C/T) in *POMC* 3’UTR.

AAs: African Americans; EAs: European Americans.

SD: substance (alcohol, cocaine, opioid and/or marijuana) dependence; AD: alcohol dependence; CD: cocaine dependence; OD: opiate dependence; MjD: marijuana dependence.

*P*_obs_: observed *P* values calculated by Chi-Square tests; *P*_emp_: empirical *P* values using 10,000 permutations; *P*_adj_: *P* values obtained from logistic regression analysis and adjusted by sex, age, BMI, and ancestry proportion; OR: odds ratio; 95% CI: 95% confidence interval.
