# Supplementary material for: Identification of POMC Exonic Variants Associated with Substance Dependence and Body Mass Index
Source: PLoS One. 2012 Sep 17;7(9):e45300. doi: 10.1371/journal.pone.0045300 (PMC3444488; doi:10.1371/journal.pone.0045300)
Supplement: Table S5 — Haplotype association of two POMC common variants (rs10654394-rs1042571) and substance dependence (SD). (DOC) [file pone.0045300.s006.doc]

**Table S5.** Haplotype association of two *POMC* common variants (rs10654394-rs1042571) and substance dependence (SD)

| **Trait** | **Race** | **HAPLOTYPE** | **Frequency** | **χ2** | ***P_obs_*** | ***P_emp_*** | **STAT** | ***P_adj_*** | **OR** |
| --- | --- | --- | --- | --- | --- | --- | --- | --- | --- |
|  |  |  | **(case/control)** |  |  |  |  |  |  |
| SD | AAs | 9 bp Del -T | 0.106/0.134 | 0.843 | 0.359 | 0.376 | 0.434 | 0.51 | 0.82 |
| SD | AAs | 9 bp Ins -C | 0.273/0.279 | 0.021 | 0.884 | 0.899 | 0.121 | 0.728 | 0.93 |
| SD | AAs | 9 bp Del -C | 0.621/0.587 | 0.55 | 0.458 | 0.429 | 0.708 | 0.40 | 1.19 |
| SD | EAs | 9 bp Del -T | 0.200/0.202 | 0.003 | 0.953 | 0.961 | 0.003 | 0.96 | 0.99 |
| SD | EAs | 9 bp Ins -C | 0.067/0.048 | 0.868 | 0.352 | 0.373 | 0.721 | 0.396 | 1.39 |
| SD | EAs | 9 bp Del -C | 0.733/0.750 | 0.191 | 0.662 | 0.66 | 0.185 | 0.667 | 0.91 |
| SD | AAs+EAs | 9 bp Del -T | 0.153/0.174 | 0.791 | 0.374 | 0.381 | 0.255 | 0.614 | 0.91 |
| SD | AAs+EAs | 9 bp Ins -C | 0.17/0.144 | 1.223 | 0.269 | 0.299 | 0.031 | 0.86 | 0.97 |
| SD | AAs+EAs | 9 bp Del -C | 0.677/0.682 | 0.024 | 0.876 | 0.895 | 0.328 | 0.567 | 1.09 |
|  |  |  |  |  |  |  |  |  |  |
| AD | AAs | 9 bp Del -T | 0.089/0.137 | 2.24 | 0.135 | 0.172 | 1.13 | 0.288 | 0.71 |
| AD | AAs | 9 bp Ins -C | 0.254/0.281 | 0.359 | 0.549 | 0.558 | 0.358 | 0.549 | 0.86 |
| AD | AAs | 9 bp Del -C | 0.658/0.582 | 2.319 | 0.128 | 0.107 | 1.91 | 0.167 | 1.37 |
| AD | EAs | 9 bp Del -T | 0.188/0.203 | 0.186 | 0.667 | 0.662 | 0.218 | 0.641 | 0.89 |
| AD | EAs | 9 bp Ins -C | 0.073/0.048 | 1.282 | 0.258 | 0.276 | 0.992 | 0.319 | 1.50 |
| AD | EAs | 9 bp Del -C | 0.740/0.748 | 0.046 | 0.83 | 0.835 | 0.022 | 0.881 | 0.97 |
| AD | AAs+EAs | 9 bp Del -T | 0.139/0.177 | 2.347 | 0.126 | 0.133 | 0.911 | 0.34 | 0.83 |
| AD | AAs+EAs | 9 bp Ins -C | 0.162/0.142 | 0.683 | 0.409 | 0.423 | 0.062 | 0.804 | 0.95 |
| AD | AAs+EAs | 9 bp Del -C | 0.699/0.681 | 0.33 | 0.566 | 0.541 | 1.01 | 0.316 | 1.17 |
|  |  |  |  |  |  |  |  |  |  |
| CD | AAs | 9 bp Del -T | 0.114/0.137 | 0.425 | 0.514 | 0.541 | 0.195 | 0.659 | 0.87 |
| CD | AAs | 9 bp Ins -C | 0.297/0.285 | 0.070 | 0.791 | 0.789 | 0.068 | 0.794 | 1.06 |
| CD | AAs | 9 bp Del -C | 0.589/0.579 | 0.038 | 0.845 | 0.833 | 0.013 | 0.910 | 1.02 |
| CD | EAs | 9 bp Del -T | 0.202/0.202 | 0.000 | 0.989 | 0.982 | 0.000 | 0.988 | 1.00 |
| CD | EAs | 9 bp Ins -C | 0.082/0.048 | 1.938 | 0.164 | 0.181 | 1.41 | 0.235 | 1.67 |
| CD | EAs | 9 bp Del -C | 0.716/0.750 | 0.538 | 0.463 | 0.477 | 0.454 | 0.500 | 0.84 |
| CD | AAs+EAs | 9 bp Del -T | 0.153/0.175 | 0.69 | 0.406 | 0.422 | 0.155 | 0.694 | 0.92 |
| CD | AAs+EAs | 9 bp Ins -C | 0.202/0.145 | 4.423 | 0.035 | 0.048 | 0.227 | 0.634 | 1.1 |
| CD | AAs+EAs | 9 bp Del -C | 0.645/0.679 | 1.013 | 0.314 | 0.332 | 0.002 | 0.967 | 0.99 |
|  |  |  |  |  |  |  |  |  |  |
| OD | AAs | 9 bp Del -T | 0.123/0.134 | 0.03 | 0.864 | 0.809 | 0.039 | 0.843 | 1.12 |
| OD | AAs | 9 bp Ins -C | 0.342/0.279 | 0.537 | 0.464 | 0.447 | 0.333 | 0.564 | 1.27 |
| OD | AAs | 9 bp Del -C | 0.535/0.587 | 0.307 | 0.58 | 0.604 | 0.465 | 0.495 | 0.76 |
| OD | EAs | 9 bp Del -T | 0.214/0.202 | 0.052 | 0.82 | 0.835 | 0.083 | 0.774 | 1.11 |
| OD | EAs | 9 bp Ins -C | 0.071/0.048 | 0.626 | 0.429 | 0.424 | 1.02 | 0.313 | 1.72 |
| OD | EAs | 9 bp Del -C | 0.714/0.750 | 0.377 | 0.539 | 0.55 | 0.652 | 0.419 | 0.77 |
| OD | AAs+EAs | 9 bp Del -T | 0.185/0.174 | 0.067 | 0.796 | 0.797 | 0.015 | 0.901 | 1.04 |
| OD | AAs+EAs | 9 bp Ins -C | 0.155/0.144 | 0.087 | 0.768 | 0.775 | 0.579 | 0.447 | 1.28 |
| OD | AAs+EAs | 9 bp Del -C | 0.660/0.682 | 0.188 | 0.665 | 0.686 | 0.454 | 0.501 | 0.85 |
|  |  |  |  |  |  |  |  |  |  |
| MjD | AAs | 9 bp Del -T | 0.119/0.134 | 0.129 | 0.72 | 0.749 | 0.005 | 0.946 | 0.98 |
| MjD | AAs | 9 bp Ins -C | 0.209/0.279 | 1.718 | 0.19 | 0.222 | 1.79 | 0.181 | 0.67 |
| MjD | AAs | 9 bp Del -C | 0.671/0.587 | 2.025 | 0.155 | 0.137 | 1.92 | 0.166 | 1.47 |
| MjD | EAs | 9 bp Del -T | 0.145/0.202 | 1.286 | 0.257 | 0.246 | 0.994 | 0.319 | 0.65 |
| MjD | EAs | 9 bp Ins -C | 0.053/0.048 | 0.028 | 0.866 | 0.81 | 0.002 | 0.964 | 1.03 |
| MjD | EAs | 9 bp Del -C | 0.803/0.750 | 0.918 | 0.338 | 0.337 | 0.748 | 0.387 | 1.38 |
| MjD | AAs+EAs | 9 bp Del -T | 0.130/0.174 | 1.854 | 0.173 | 0.185 | 0.62 | 0.431 | 0.81 |
| MjD | AAs+EAs | 9 bp Ins -C | 0.141/0.144 | 0.007 | 0.932 | 0.94 | 2.16 | 0.142 | 0.67 |
| MjD | AAs+EAs | 9 bp Del -C | 0.729/0.682 | 1.335 | 0.248 | 0.235 | 3.41 | 0.065 | 1.50 |

rs10654394: a 9-bp insertion/deletion polymorphism (-/AGCAGCGGC) in *POMC* exon 4; rs1042571: a SNP marker (C/T) in *POMC* 3’UTR.

AAs: African Americans; EAs: European Americans.

SD: substance (alcohol, cocaine, opioid and/or marijuana) dependence; AD: alcohol dependence; CD: cocaine dependence; OD: opiate dependence; MjD: marijuana dependence.

*P*_obs_: observed *P* values calculated by Chi-Square tests; *P*_emp_: empirical *P* values using 1,000 Monte Carlo permutations *P*_adj_: *P* values adjusted by sex, age, BMI and race using logistic regression analyses.
